# Intensivpflege in Zeiten der COVID-19 Pandemie: Zur Frage des Verhältnisses von Fürsorge und Selbstsorge

**DOI:** 10.1007/s00481-021-00606-5

**Published:** 2021-01-27

**Authors:** Eva Kuhn, Anna-Henrikje Seidlein

**Affiliations:** 1 Sektion Global Health, Institut für Hygiene und Öffentliche Gesundheit, Universitätsklinikum Bonn, Venusberg-Campus 1, 53127 Bonn, Deutschland; 2 Institut für Ethik und Geschichte der Medizin, Universitätsmedizin Greifswald, Ellernholzstr. 1–2, 17487 Greifswald, Deutschland

**Keywords:** COVID-19, Pandemie, Intensivpflege, Care Ethik, Fürsorge, Selbstsorge, Ethikkodex, COVID-19, Pandemic, Intensive care nursing, Care ethics, Care, Self-care, Code of ethics

## Abstract

Die COVID-19 Pandemie stellt eine beträchtliche Herausforderung für die Kapazität und Funktionalität der Intensivversorgung dar. Dies betrifft nicht nur Ressourcen, sondern vor allem auch die körperlichen und psychischen Grenzen von Pflegefachpersonen. Der Frage, wie sich Fürsorge und Selbstsorge von Pflegefachpersonen auf Intensivstationen im Rahmen der COVID-19 Pandemie zueinander verhalten, wurde bislang im öffentlichen und wissenschaftlichen Diskurs keine Aufmerksamkeit geschenkt. Der vorliegende Beitrag reflektiert dieses Verhältnis mit Hilfe des Ethikkodex des International Council of Nurses, unter besonderer Berücksichtigung der Prinzipienethik und der Care-Ethik nach Joan Tronto und zeigt einen Korridor ethisch vertretbarer Pflege auf.

Die Arbeit leistet damit einen wichtigen Beitrag zu einer differenzierten ethischen Betrachtung der Rechte und Verantwortlichkeiten von Pflegefachpersonen als moralischen Akteuren innerhalb des Pandemiegeschehens in Deutschland. Damit schafft er eine erste Voraussetzung für einen breiten gesellschaftlichen und politischen Diskurs, der über die Pandemie hinaus dringend notwendig ist, um die Situation der Pflegefachpersonen und der Gepflegten nachhaltig zu verbessern.

## Abstract (en)

**Definition of the problem:**

The COVID-19 pandemic poses a considerable challenge to the capacity and functionality of intensive care. This concerns not only resources but, above all, the physical and psychological boundaries of nursing professionals. The question of how care for others and self-care of nurses in intensive care units are related to each other in the context of the COVID-19 pandemic has not been addressed in public and scientific discourse so far.

**Arguments:**

The present contribution reflects this relationship with reference to the Code of Ethics of the International Council of Nurses, particularly considering principlism and the Care Ethics according to Joan Tronto. As a result, it shows a corridor of ethically justifiable care with several borders, above all: (1) self-care must not be given up completely for the benefit of care for others, and (2) a categorical subordination of care for others to self-care is ethically not justifiable.

**Conclusion:**

The article makes an important contribution to a differentiated ethical consideration of the rights and responsibilities of intensive care nurses as moral actors within the pandemic in Germany. It, thus, provides a first starting point for a broad social and political discourse which is urgently needed not only during but also after the pandemic in order to improve the situation of intensive care nurses and those who are cared for.

## Einleitung

Die Erkrankung COVID-19 ist im März 2020 vom Generaldirektor der Weltgesundheitsorganisation (WHO) zur Pandemie erklärt worden. Intensivbetten stellen eine Schlüsselressource für die Behandlung der COVID-19 Patient*innen dar, da schwere Verläufe mit der Notwendigkeit (nicht-)invasiver Beatmung einhergehen. Kennzeichnend für die COVID-19 Pandemie ist, so zeigen es die bisherigen Erfahrungen aus anderen Ländern, dass insbesondere im Hinblick auf Intensivbetten der Bedarf zeitweilig um ein Vielfaches größer ist als die vorhandenen Kapazitäten (Grasselli et al. 2020). Diese – möglichen – Engpässe klinischer Versorgung werfen eine Reihe ethischer Fragen auf, darunter prominent Fragen der (Verteilungs‑)Gerechtigkeit im Zusammenhang mit notwendigen Allokationsentscheidungen über die intensivmedizinische Behandlung in Triagesituationen (Deutscher Ethikrat 2020; DIVI et al. 2020; Stoecker 2020). Deutschland hält im Vergleich zu anderen EU-Ländern einen sehr hohen Anteil an Intensivkapazität vor (Statistisches Bundesamt 2018, S. 68). Nichtsdestotrotz arbeitet auch Deutschland seit März daran, die Intensivkapazitäten aufzustocken, um den Hilfsbedarf weiterhin decken zu können. Auch wenn entsprechend des „Grobkonzepts Infrastruktur Krankenhaus“ vom 17. März 2020, welches eine Verdopplung der Intensivkapazitäten vorsieht, räumliche und medizinisch-technische Herausforderungen schrittweise gemeistert werden, so darf nicht in Vergessenheit geraten, dass die Intensivversorgung nicht nur im Hinblick auf die begrenzten finanziellen, räumlichen und instrumentellen Ressourcen an die Grenzen ihrer Kapazität und Funktionalität kommt. Vielmehr riskiert das medizinische Personal auch seine körperliche (ICN 2020b) und psychische Gesundheit (Neto et al. 2020), wenn den gesteigerten materiellen Ressourcen keine adäquaten personellen Ressourcen (im Sinne einer 1:1 Nurse-to-Patient-Ratio) entgegengesetzt werden können. Unzureichende Personalschlüssel verschlechtern die Adhärenz mit hygienischen Standards (WHO 2009, S. 6) und gehen so auch mit einer erhöhten Infektionsgefahr für die Pflegenden selbst einher. Zudem wird „care left undone“ bzw. „missed care“ befördert, was zu „moral distress“ und weiteren negativen psychischen Konsequenzen auf Seiten der Pflegenden führen kann (Suhonen et al. 2018). Insbesondere Pflegefachpersonen (PP)^1^ haben weiterhin engen und häufigen Kontakt mit Patient*innen. Doch anders als beispielsweise Ärzt*innen (BÄK 2019) wurde PP im öffentlichen und wissenschaftlichen Diskurs bislang wenig Beachtung geschenkt. Vielmehr ist auch im Jahr der Pflegenden und Hebammen die Feststellung, dass PP „oft unter herausfordernden Bedingungen zu arbeiten [haben]: unterschätzt – unterbesetzt – überlastet“ (DBfK 2020a), aktueller denn je. Gleichzeitig kommt ihnen als der größten Berufsgruppe im Gesundheitswesen jedoch eine zentrale Rolle und „grundlegende professionelle Verantwortung“ (ICN 2012) bei der kurativen und palliativen Begleitung COVID-19-Erkrankter auf Intensivstationen (ITS) zu.

Neben Fragen danach, wie Pflege-Ressourcen bei ausgelasteten oder gar überlasteten ITS rationiert werden sollen, werfen Maßnahmen wie die Aussetzung der Pflegepersonaluntergrenzen (PpU) und Lockerung der Vorschriften zur persönlichen Schutzausrüstung (PSA) Licht auf eine andere Überlegung: Wie sollten sich in der COVID-19 Pandemie Fürsorge und Selbstsorge von ITS-PP zueinander verhalten? Hintergrund dieser Überlegung ist die Annahme, dass „An der Selbstsorge […] Andere [sic!] direkt oder indirekt beteiligt“ sind (Schnell 2017, S. 50). Denn angesichts der Leiblichkeit, Endlichkeit und daraus resultierender Vulnerabilität der Menschen, sind Fürsorge und Selbstsorge grundlegende Modi menschlichen Daseins. Diese müssen deshalb folgerichtig als einander bedingend und beeinflussend verstanden werden und somit auch gemeinsam betrachtet werden. Es geht also nicht um ein „Entweder-oder“, sondern um ein „Sowohl-als-auch“, wobei „sowohl – als auch“ nicht heißen muss, dass Selbst- und Fürsorge dieselbe Aufmerksamkeit gewidmet wird, sondern je neu ihr Verhältnis zueinander zu bestimmen ist. Die damit verbundene Abwägung geht über eine rein deskriptive Erfassung einer konkreten Situation hinaus. Vielmehr drängt sich die Frage nach zugrundeliegenden normativen Kriterien für eine Verhältnisbestimmung zwischen Für- und Selbstsorge auf. Eine vergleichbare Frage, nämlich wie viel persönliches Risiko und Selbstaufopferung von PP gerechtfertigterweise erwartet werden kann, wurde bereits 2014 von Johnstone und Turale als Forschungslücke identifiziert, bislang jedoch nicht näher betrachtet.

Auch über das Pandemiegeschehen hinaus dient das Füllen dieser Forschungslücke einer Perspektiverweiterung der Ethik im Gesundheitswesen: Die Fokussierung der Prinzipienethik auf die Person des*der Patient*in, die sich in den Prinzipien Fürsorge, Nicht-Schaden und Respekt der Autonomie widerspiegelt, wird durch einen Blick auf das Care-Netzwerk ergänzt und dadurch die Umstände fürsorglichen Handelns verstärkt in den Blick genommen.

Entsprechend wird im vorliegenden Beitrag die Situation von PP, die in direktem Patient*innenkontakt auf der ITS^2^ stehen, als moralischen Akteuren innerhalb des Pandemiegeschehens in Deutschland vor dem Hintergrund aktueller politischer Entscheidungen, Problemlagen und Empfehlungen ethisch reflektiert. Damit sollen weder diese auf komplexen Abwägungen beruhenden Entscheidungen diskreditiert werden, noch will die Fokussierung auf PP die mit der Pandemie einhergehenden Belastungen und ethischen Konflikte anderer Gesundheitsprofessionen wie Ärzt*innen schmälern (dazu u. a. Kannampallil et al. 2020). Vielmehr erlaubt sie, „die ohnehin fragile professionelle Integrität angesichts schwieriger Arbeitsbedingungen, teilweise inkonsistenter politischer Forderungen und hoher gesellschaftlicher Erwartungen“ (AEM 2020, S. 4) sowie „grundlegende Gerechtigkeitsprobleme“ (Bobbert 2019, S. 290; vgl. dazu auch Dichter et al. 2020) beruflicher Pflege in das Zentrum zu stellen. Die ethische Frage nach dem Recht, sich zu schützen und für sich selbst zu sorgen, im Verhältnis zu der Verantwortung zu pflegen, wird dabei aus der Perspektive der Pflege gestellt. Im Fokus stehen folglich nicht die gesellschaftlichen Ansprüche der Allgemeinbevölkerung gegenüber den beruflich Pflegenden, sondern deren professionelle Verantwortung und letztlich Selbstverpflichtung.

Eine gesonderte Betrachtung der Situation von PP mit Führungsaufgaben (bspw. Pflegedienstleitung) kann in diesem Beitrag nicht geleistet werden. Grundlage der Verhältnisbestimmung von Selbst- und Fürsorge sind der ICN-Ethikkodex, mit besonderer Berücksichtigung der Prinzipienethik, sowie der moralphilosophische Ansatz der Care Ethik. Der Beitrag schließt mit einer Einordnung dieser Abwägung in den organisationalen und politischen Kontext.

## Hintergrund

### Pandemie-unabhängige Situation von PP^3^

Die Situation der (ITS-)Pflege stellt sich als circulus vitiosus dar: In Deutschland herrscht bereits als Ausgangssituation eine im Vergleich niedrigere Nurse-to-Patient-Ratio als in anderen EU-Ländern (Aiken et al. 2014). Die Arbeitsbelastung steigt damit in einer ohnehin schon sehr belastenden Arbeitsumgebung weiter und begünstigt eine hohe Fluktuation von der Station oder sogar komplett aus der Pflege. Der von der DIVI (2010) empfohlene Personalschlüssel (eine PP betreut pro Schicht zwei Patient*innen) kann aufgrund von Personalmangel oft nicht gewährleistet werden (Karagiannidis et al. 2019). Mittlerweile führt der Mangel an qualifizierten ITS-PP regelhaft zu Sperrungen von Betten auf vielen ITS (Karagiannidis et al. 2018; Isfort 2017). Parallel lässt sich eine Zunahme von Teilzeitarbeit verzeichnen (Statistisches Bundesamt 2018, S. 8), die nicht zuletzt auch, aber nicht ausschließlich, darauf zurückzuführen ist, dass der Anteil der beschäftigten Frauen in der Pflegeprofession nach wie vor sehr hoch ist (BA 2019). Frauen übernehmen überdurchschnittlich häufig zeitgleich auch andere Sorgearbeit in der Familie und reduzieren dafür ihre Erwerbstätigkeit (DZA 2018, S. 21). Dies bringt sie in eine „Sandwich-Position“ mit dreifacher Verpflichtung (Kinderbetreuung, Pflege der Angehörigen, Ausübung der Profession), die sich in Extremsituationen wie einer Pandemie noch verschärft (Malm et al. 2008, S. 16). Es müssen folglich mindestens zwei Dimensionen konkurrierender Fürsorge für andere *und* Selbstsorge ausdifferenziert werden, die in diesem Zusammenhang relevant sind: 1. Fürsorge für Patient*innen und Selbstsorge von PP im Kontext der Berufsausübung sowie 2. Fürsorge für An- und Zugehörige mit PP in der Rolle informell Pflegender. Diese Situation ist insofern insbesondere für PP kennzeichnend, als dass ein beachtlicher Anteil beruflich Pflegender zeitgleich mehrere Sorgeaufgaben übernimmt, da sie aufgrund ihrer spezifischen Expertise auch als informell Pflegende herangezogen werden (Ward-Griffin et al. 2015).

### Pandemie-bedingte Herausforderungen

In dieser bereits multifaktoriell belastenden Ausgangslage der (ITS-)PP im Krankenhaus soll nun das Augenmerk auf vier aktuelle Entscheidungen und Problemlagen im Rahmen der COVID-19 Pandemie gelenkt werden, die für diese PP von praktischer und auch moralischer Relevanz sind. Diese Entscheidungen sind selbst unter Abwägung verschiedener Rechtsgüter und Gesichtspunkte wie der öffentlichen Gesundheit, Fairness und Solidarität (Leopoldina 2020, S. 10ff.) und zumeist unter hohem Zeitdruck aufgrund der äußerst dynamischen Entwicklung getroffen worden. Anders als in der aktuellen Public Health Diskussion (Gostin et al. 2020; Kompetenznetz Public Health zu COVID-19/AG Ethik 2020) steht im Folgenden nicht die Frage nach der Billigkeit und Angemessenheit dieser Entscheidungen im Vordergrund. Vielmehr werden diese exemplarisch für die Bestimmung des Korridors ethisch vertretbarer Pflege herangezogen. Diesen zukünftig bei einer Anpassung des „crisis standard of care“ zu berücksichtigen, ist Aufgabe der jeweiligen Entscheidungsträger*innen. Um Verantwortungsdiffusion zu vermeiden, ist hierbei notwendigerweise die Differenz zwischen kurzfristig beeinflussbaren Faktoren (wie z. B. Priorisierung ökonomischer Interessen der Krankenhäuser) und solchen, die von den Akteur*innen akut nicht beeinflussbar sind (z. B. hohes Patient*innenaufkommen, andauernde Personalknappheit), zu berücksichtigen. Eine solche Trennung ist jedoch besonders für Pandemie-spezifische Problemlagen nicht durchzuhalten.

#### Pflegepersonaluntergrenzen-Verordnung (PpUGV)

Mit Inkrafttreten der PpUGV gelten seit Januar 2019 (BGBl Teil I Nr. 37) nun vorerst für vier sogenannte „pflegesensitive Bereiche“^4^ verbindliche PpU. Für ITS als einem dieser Bereiche gilt derzeit, dass in den Tagesschichten eine PP maximal 2,5 Patient*innen, im Nachtdienst maximal 3,5 Patient*innen betreuen darf. Trotz begründeter Kritik können die gesetzlichen PpU als ein erster Schritt in die richtige Richtung mit Blick auf Pflegequalität und Patient*innensicherheit betrachtet werden. Im Rahmen der COVID-19 Pandemie wurde mit Wirkung vom 1. März 2020 als einer der sieben Punkte des Maßnahmenpaketes zur „Entlastung von Pflegekräften“ innerhalb der Bundesregierung die Aussetzung der PpU beschlossen (Bundesregierung 2020). Zwar wurde zum 1. August 2020 die PpU (als beeinflussbarem Faktor) in der Intensivpflege wiedereingesetzt, dennoch bleibt die Nurse-to-Patient-Ratio angesichts der bereits vor der COVID-19 Pandemie bestehenden „Aufweichung“ selbiger in der täglichen Praxis (DBfK 2020b) und durch personelle Engpässe (verstärkt u. a. durch Anordnung häuslicher Quarantäne und die beginnende Grippesaison) faktisch weiterhin unter der PpU, ist also unbeeinflussbar. Zunehmend wird thematisiert, dass nicht die Beatmungsgeräte, sondern die verfügbaren Fachkräfte über die tatsächliche Intensivkapazität bestimmen (Tagesschau.de 2020). Eine Aussetzung der PpU steht deshalb mit Datum vom 23.10.2020 wieder zur Debatte (Ärzteblatt.de 2020).

#### Zwangsverpflichtung von PP

Gesetzesentwürfe und Beschlüsse aus dem Frühjahr 2020 greifen darüber hinaus das Thema Zwangsverpflichtung von medizinischem Fachpersonal auf: Der Epidemie-Gesetzentwurf für Nordrhein-Westfalen sowie die Reform des Bundesgesetzes zur Verhütung und Bekämpfung von Infektionskrankheiten beim Menschen sahen eine Zwangsrekrutierung vor. Eine explizite „Zwangsverpflichtung“ für die Gesundheitsprofessionen lässt sich im neugefassten Infektionsschutzgesetz (BGBl Teil I 2020 Nr. 14) zwar nicht mehr finden, dennoch bleibt die Frage nach der Auslegung im Hinblick auf die Möglichkeit „durch Rechtsverordnung […] Maßnahmen zur Aufrechterhaltung der Gesundheitsversorgung in […] Krankenhäusern […] in Abweichung von bestehenden gesetzlichen Vorgaben vorzusehen“ (§ 5 Abs. 2 Nr. 7 IfSG).

#### Empfehlungen zum Management von PP als COVID-19 Kontaktpersonen

Zwischen dem Auftreten von Symptomen und der Ansteckungsgefahr für die Kontaktpersonen muss nicht zwangsläufig ein Zusammenhang bestehen. Ein Großteil der Erkrankungen verläuft symptomlos oder mit sehr milden Symptomen. Darüber hinaus ist derzeit noch unklar, wie lange Träger*innen des Virus ansteckend sind.

Die Empfehlungen des Robert Koch-Instituts (RKI) zum Management von Kontaktpersonen unter medizinischem Personal (RKI 2020b) wurden seit Pandemiebeginn gelockert. Vor dem Hintergrund der zwei konfligierenden Ziele Infektionsschutz und Gewährleisten der akutmedizinischen Versorgung unterscheiden die Empfehlungen zwischen Situationen mit und ohne relevantem Personalmangel. Sie sehen eine mögliche Verkürzung der häuslichen Quarantäne vor, sowie die Option, dass PP ohne Symptome weiterhin in der direkten Patient*innenversorgung tätig bleiben.

#### Mangel an PSA

Die Empfehlungen des RKI kommen jedoch mit einer weiteren Problemlage einher. Es gehört zu den grundlegenden Pflichten des Arbeitgebers, „die erforderlichen Maßnahmen des Arbeitsschutzes unter Berücksichtigung der Umstände zu treffen, die Sicherheit und Gesundheit der Beschäftigten bei der Arbeit beeinflussen“ (§ 3 Abs. 1 S. 1 Arbeitsschutzgesetz). Die Umstände der COVID-19 Pandemie bringen es jedoch mit sich, dass ein eklatanter Mangel an PSA durch Lieferengpässe v. a. von chirurgischem Mundschutz, FFP2/FFP3-Masken, Schutzvisieren und Desinfektionsmittel zu verzeichnen ist (BMG 2020). Aufgrund der Übertragung von SARS-CoV‑2 durch Tröpfcheninfektion haben PP auf ITS ein sehr hohes Ansteckungsrisiko, da sie regelhaft bei der Ausübung pflegerischer Tätigkeiten Aerosolen ausgesetzt sind. Dabei wissen PP nicht immer, ob die Patient*innen Träger des Virus sind, sodass eine Behandlung auf der ITS, bei der primär eine andere Diagnose im Vordergrund steht, denkbar ist. Dennoch sind die Empfehlungen zu Hygienemaßnahmen im Verlauf der COVID-19 Pandemie gelockert bzw. herabgesetzt worden.^5^

### Der „crisis standard of care“

Der zu Pandemiebeginn wirksame und in weiten Teilen auch noch im Herbst 2020 gültige „crisis standard of care“, d. h. der aufgrund von Einschränkungen bei Versorgung, Personal u. a. mögliche Versorgungsgrad während einer Krise (IOM 2009, S. 112), lässt sich für ITS-PP in Deutschland entsprechend unter den Schlagworten Aussetzung sowie Missachtung von PpU, Mangel an PSA, Verkürzung bzw. Verzicht der Quarantäne sowie letztlich auch Aufhebung von Beschlüssen der Selbstverwaltung durch das Bundesministerium für Gesundheit („Zwangsverpflichtung“) zusammenfassen.

Hinzu kommt – und daraus resultiert, wie empirische Erhebungen im Zusammenhang mit anderen Pandemien gezeigt haben, dass PP um ihre eigene Gesundheit und die ihrer Familie fürchten, an die sie die Infektion weitertragen könnten (Ehrenstein et al. 2006). Schließlich sind PP besonders lange mit (potenziell) infizierten Patient*innen in direktem Kontakt und durch ihre Pflegetätigkeiten einer besonders hohen Viruslast ausgesetzt. Für diese Situationskonstellation muss deshalb festgestellt werden, dass sie *außergewöhnliche* Risiken mit sich bringt, die über *allgemeine* berufliche Risiken in der Pflege hinausgehen und Fragen des angemessenen Umgangs mit dieser besonderen Risikosituation aufwerfen (Wolff et al. 2020). Auch im Rahmen der COVID-19 Pandemie infizieren sich PP und versterben^6^. Zudem können sie durch ihre individuelle Prädisposition (z. B. chronische Vorerkrankungen wie Asthma) ein besonderes Risiko für einen schweren Krankheitsverlauf tragen. Diese Tatsachen lassen auch PP erwägen, sich in Pandemiesituationen aus dem Beruf zurückzuziehen (Shiao et al. 2007).

## Ethik in der Pflege und für die Pflege

Ethik in der Pflege und für die Pflege folgt nicht einem universalen Ansatz, d. h. sie bedient sich verschiedener theoretischer Zugänge.^7^ Über die Prinzipienethik als kleinsten gemeinsamen Nenner aller Gesundheitsberufe hinaus (Riedel et al. 2017, S. 163), besteht lediglich ein Minimalkonsens darin, *dass* PP eine Verantwortung für ihre Patient*innen haben (Lachmann 2012, S. 114). Umfang und Ausmaß dieser Verantwortung sind jedoch abhängig von der spezifischen Situation und der im Einzelfall vorgenommenen Abwägung der tangierten moralischen Prinzipien. Außerdem kommen verschiedene Begründungsquellen für die Pflicht von PP zur Behandlung im Fall einer ernsten Infektionskrankheit, die sie selbst einem bedeutenden Risiko aussetzt, in Betracht. Eine solche Begründung kann auf der ausdrücklichen oder impliziten Zustimmung fußen, der (teilweise auch staatlich finanzierten) Fachausbildung und dem Gedanken der Reziprozität bzw. eines Gesellschaftsvertrags. Außerdem finden sich ethische Begründungen insbesondere in Gelöbnissen sowie Kodizes der Profession (Malm et al. 2008). Letztere Begründungsquelle, professionsethische Kodizes, ist vorliegend ein Ausgangspunkt. Die anderen vier Begründungsquellen werden an dieser Stelle nicht näher untersucht, da – so auch von Malm et al. (2008, S. 7ff.) ausgeführt – PP bei Vertragsschluss im Regelfall bislang weder auf das Risiko einer Pandemie aufmerksam gemacht wurden noch dieses mitbedacht haben. Eine solche, für einen gültigen Arbeitsvertrag nicht verpflichtende Klausel würde bedeuten, dass PP mit Vertragsunterzeichnung, abhängig von der Formulierung, explizit einwilligen, auch hochinfektiöse Patient*innen, bspw. im Rahmen einer Pandemie, zu behandeln und sich dazu folglich vertraglich zu verpflichten. Ebenso greift die Begründungsquelle der Fachausbildung zu kurz, da im Rahmen der COVID-19 Pandemie auch verstärkt PP auf ITS eingesetzt werden, die für diese nicht primär ausgebildet und qualifiziert sind, sondern im Rahmen von Personalumverteilungskonzepten dort eingesetzt werden sollen (DIVI 2020). Auch die Begründung einer Sorge*pflicht* unter Rekurs auf Reziprozität, d. h. einen Vertrag zwischen Gesellschaft und PP, ist zu unspezifisch. Eine solche kann nur Aussagen über die Gruppe der PP als ganze machen, nicht aber alle Aspekte und Prinzipien für eine Abwägung im Einzelfall generieren (Malm et al. 2008, S. 11). Anders als z. B. Ärzt*innen, werden beruflich Pflegende nach wie vor als „(Semi‑)Profession“ diskutiert (Kälble 2017). Aus ihrem Status als (Semi‑)Profession erwachsende Vorteile sind demnach weniger durch besondere Vorteile in (im)materieller Anerkennung gekennzeichnet als vielmehr durch ein besonderes Vulnerabilitätsniveau, das sich aus den vorherrschenden Meinungen über ihren Berufsstand und den unzulänglichen Rahmenbedingungen für ihre Arbeit ergibt (Schrems 2020, S. 35ff.). Darüber hinaus kann – selbst bei Anerkennung des Professionsstatus – allein durch die Zugehörigkeit einzelner PP zur Gruppe der beruflich Pflegenden nicht davon ausgegangen werden, dass für *einzelne* PP aus dem damit verbundenen Ansehen tatsächlich Vorteile erwachsen (vgl. dazu auch Malm et al. 2008, S. 12).

Im Folgenden wird deshalb exemplarisch der ICN-Ethikkodex für Pflegende unter besonderer Berücksichtigung der Prinzipienethik als Anhaltspunkt für dessen konkretes Verständnis beleuchtet und durch den moralphilosophischen Ansatz der Care-Ethik nach Joan Tronto komplementiert. Diese Auswahl begründet sich durch die Konzentration des ICN-Ethikkodexes auf professionelle Verantwortung. Der ICN-Ethikkodex und die Prinzipienethik legen in Teilen dieselben ethischen Verhaltensweisen zugrunde, wenn auch mit anderer Begründung und Tiefe. Die Prinzipienethik kann in diesem Sinne den ICN-Ethikkodex substantiieren. Sie dient folglich hier als Vehikel für die Anwendung des ICN-Ethikkodex auf die konkrete Situation. Tronto ihrerseits fokussiert ebenso auf eine verantwortungsbasierte Ethik (Tronto 1993, S. 131ff.), in der sie Verantwortung – im Unterschied zu Ansätzen, die auf Pflichten basieren – als kontextrelatives Handlungsprinzip und relationales Konzept betrachtet. Die von ihr entwickelten vier Komponenten des Caring eignen sich besonders, diese Relationalität im Care-Prozess allgemein sowie im Pflegeprozess im Besonderen nachzuvollziehen. Entsprechend gibt Trontos Ansatz Anhaltspunkte für dem pflegerischen Handeln zugrundliegende moralische Prinzipien, die auf den Fall von ITS-Pflege in Zeiten der COVID-19 Pandemie angewendet und gegeneinander abgewogen werden.

### ICN-Ethikkodex für Pflegende, unter besonderer Berücksichtigung der Prinzipienethik

Auch wenn Malm et al. kritisch anmerken, dass Kodizes erstens von den Adressat*innen stärker als lediglich symbolisch und weniger als handlungsleitend angesehen werden und zweitens sehr allgemein und mit großem Interpretationsspielraum formuliert sind (Malm et al. 2008, S. 13, 16), geben sie dennoch Aufschluss über die grundlegenden Werte und Prinzipien der entsprechenden Profession. Verstanden als Bezugssystem, das professionsethisches Handeln leitet, ist ein Ethikkodex ein Instrument der berufsständischen Selbstverwaltung (Ruderman et al. 2006). Werden Kodizes Teil der Berufsordnung (z. B. im Rahmen von Pflegekammergesetzen), so gewinnen sie an Gesetzeskraft. Entsprechend legt auch der ICN-Ethikkodex für Pflegende^8^ „den Kern der ethischen Identität der Pflege“ nieder (Riedel et al. 2017, S. 163). Der Kodex des International Council of Nurses (ICN 2012) zeichnet sich durch den Rückbezug auf universale Werte und Prinzipien und dabei insbesondere durch seine Orientierung an den Menschenrechten aus. Dies erhöht nicht nur seine Verbindlichkeit, sondern macht ihn auch supranational anwendbar. Der Deutsche Berufsverband für Pflegeberufe (DBfK) sieht den Ethikkodex als Basis für die Berufsausübung der PP in Deutschland an und beruft sich entsprechend auch in der öffentlichen Debatte ausdrücklich auf den Kodex.^9^ Zudem wird er vom Deutschen Pflegerat als sog. Berufsethik unter beruflich Pflegenden verbreitet^10^ und ist durch Aus- und Fortbildung einem Großteil der PP in Deutschland bekannt.^11^

Nach dem ICN gilt die „grundlegende professionelle Verantwortung der Pflegenden […] dem pflegebedürftigen Menschen“. Zudem sind PP „persönlich verantwortlich und rechenschaftspflichtig für die Ausübung der Pflege“ (ICN 2012, S. 2). Die vier Aufgaben der PP sind entsprechend der Präambel die Förderung von Gesundheit, die Verhütung von Krankheit, die Wiederherstellung von Gesundheit sowie die Linderung von Leid. Der ICN-Ethikkodex selbst buchstabiert nicht aus, wie PP diese Aufgaben zu verstehen und wahrzunehmen haben. In der Literatur resoniert der Grundsatz der grundlegenden professionellen Verantwortung in der Debatte um die Pflicht zur Pflege („duty to care“), in welcher die beiden Prinzipien Fürsorge und Nicht-Schaden aus der Prinzipienethik im Zentrum stehen (Schroeter 2008, S. 3). Für die Auslegung des ICN-Ethikkodexes kann die Prinzipienethik jedoch nur Anhaltspunkte geben, da Letztere deutlich zentristischer ist als der universal angelegte ICN-Ethikkodex. Simonds und Sokol (2009) zufolge umfasst Fürsorge in einer Pandemie nicht nur die Sorge um das Wohl der Patient*innen selbst, sondern auch die Pflicht, im Interesse der An- und Zugehörigen zu handeln sowie die Verantwortung, Kolleg*innen, den Arbeitgeber, die Profession als solche sowie die Gesellschaft zu unterstützen. Dem entspricht, im Sinne des Prinzips des Nicht-Schadens, die Verpflichtung, das Risiko für Patient*innen sowie Team-Mitglieder, Familienangehörige, aber auch für sich selbst möglichst gering zu halten. Außerdem kommt PP die Pflicht zu, die weitere Ausbreitung der Infektionskrankheit durch entsprechende Maßnahmen zu verhindern. Schließlich umfasst das Prinzip des Nicht-Schadens gemäß Simonds und Sokol auch, einen möglichen Vertrauensverlust in Gesundheitsberufe sowie andere Nachteile für die Profession dadurch zu verhindern, dass PP die Behandlung und Pflege hochinfektiöser Patient*innen nicht ablehnen (Simonds und Sokol 2009, S. 305f.). Dieser Gedanke wiederum findet sich auch im ICN-Kodex, in welchem PP dazu angehalten werden, „das Vertrauen der Bevölkerung in den Pflegeberuf zu stärken“ (ICN 2012). Durch seine menschenrechtliche Begründung weit über die Prinzipienethik hinausgehend, umfasst der ICN-Kodex ethischer Verhaltensweisen auch, dass PP „auf ihre eigene Gesundheit [achten], um ihre Fähigkeit zur Berufsausübung nicht zu beeinträchtigen“ (ICN 2012, S. 3).

Folglich haben PP nicht nur eine Verantwortung gegenüber anderen, sondern auch sich selbst gegenüber. Es muss an dieser Stelle kritisch angemerkt werden, dass die Darstellung der Verantwortung respektive Pflicht zur Selbstsorge ein instrumentelles Verständnis der Gesundheit Pflegender impliziert, da es direkt an die Fähigkeit zur Pflegeübernahme geknüpft wird (Seidlein et al. 2019). Diese gerade in einer Pandemie über einen längeren Zeitraum hinweg überbordende Verantwortung für andere wie für sich selbst und die daraus resultierende fortwährende Abwägung lassen sich jedoch unter alleinigem Rekurs auf Prinzipienethik und ICN-Kodex nicht auflösen. Vielmehr ist der größere Kontext, darunter berufliche Beziehungen und soziale (Macht‑)Strukturen, in die Abwägung und Entscheidungsfindung einzubeziehen (Austin 2008).

### Care-Ethik

Den Einbezug solcher Aspekte erlaubt der moralphilosophische Ansatz der Care-Ethik. „Care“ ist dabei nicht mit der pflegerischen Tätigkeit gleichzusetzen und ebenso wenig instrumentell zu verstehen, sondern geht weit über sie hinaus. So wird Care zumeist definiert als Tätigkeit oder Tugend, die auf das Erhalten der Welt sowie auf die Befriedigung unserer eigenen Bedürfnisse wie der Bedürfnisse anderer zielt. Care-Ethik zeichnet sich folglich durch die Betonung des relationalen Moments, der Verbundenheit mit- und Abhängigkeit voneinander sowie eine Bedürfnisorientierung aus. Sie schreibt den grundlegenden Komponenten von (Sorge‑)Beziehungen moralische Bedeutung zu (Sander-Staudt 2019).

Für Tronto umfasst die Care-Ethik, einen entsprechenden Care-Habitus zu entwickeln, sich seinem Gegenüber zuzuwenden und sich selbst gleichzeitig zu fragen, wie die Care-Verantwortung bestmöglich erfüllt werden kann (Tronto 1993, S. 110, 127). Das Sorgen (*Caring*), bspw. für Patient*innen, hat ihr zufolge vier Phasen: *caring about* – *taking care of* – *caregiving* – *care receiving* (Tronto 1993, S. 165). Notwendig für erfolgreiches sowie wirkungsvolles *Caring* sind wiederum vier grundliegende Komponenten: Aufmerksamkeit (*attentiveness*) – Verantwortung (*responsibility*) – Kompetenz (*competence*) – Reaktion des*der Gepflegten (*responsiveness of the care receiver*) (Tronto 1993, S. 127).

Nur wer aufmerksam ist, kann erkennen, dass jemand – das Gegenüber oder man selbst – ein Bedürfnis hat. Zum einen ist für Tronto Ignoranz ein moralisches Übel. Zum anderen hebt sie hervor, dass es, wenn die Aufmerksamkeit auf Bedürfnisse anderer geht, wichtig ist, auch auf seine eigenen Bedürfnisse zu achten und sich nicht mit dem Gegenüber überzuidentifizieren (Tronto 1993, S. 127ff.). Die ethische Dimension der Verantwortung betont hingegen, dass *Caring* über eine Pflichterfüllung hinausgeht. Damit zusammen hängt auch die Frage nach Kompetenz, welche für Tronto insofern eine ethische Dimension hat, als dass der*die Sorgende bei eigenem fachlichen Unvermögen oder fehlenden Ressourcen dafür Sorge zu tragen hat, dass ein*e andere*r Fachkundige*r die Sorgearbeit ausführt (Tronto 1993, S. 133f.). Schließlich werden durch die vierte Dimension, die Reaktion – von Kohlen mit „Resonanz“ übersetzt (Kohlen 2018, S. 263), die ethischen Konzepte und Herausforderungen der Vulnerabilität sowie (Un‑)Gleichheit, Abhängigkeit und Machtasymmetrie in einer Care-Beziehung hervorgehoben (Tronto 1993, S. 134ff.). Außerdem verweist diese letzte Dimension auf die Wichtigkeit der Selbstsorge des*der Sorgenden: Die Reaktion des Gegenüber und damit die Reziprozität der Beziehung (Watson 2001) impliziert zugleich, dass der*die Gepflegte nicht als Mittel zum Zweck der eigenen Genugtuung „gebraucht“ und als Mittel zum Zweck der eigenen Selbstsorge stilisiert werden darf. Vielmehr ist Selbstsorge ein Teil der *Care Work* (Sorgearbeit). Selbstsorge „umfasst die Sorge um die eigene Gesundheit, Bildung und individuellen Bedürfnisse. Sorgearbeit für sich selbst ist im Beruf, im Familien- und Freundeskreis sowie in anderen Bereichen notwendig, um das eigene Arbeitsvermögen aufrecht zu erhalten“ (Kohlen 2018, S. 268). Dadurch wird deutlich, dass Selbstsorge weder isoliert betrachtet und absolut gesetzt, noch verkürzt allein für die Aufrechterhaltung der beruflichen (Pflege‑)Tätigkeit instrumentalisiert werden kann. Vielmehr ist nicht nur bei Care, sondern auch bei Self-Care das relationale Moment und damit relative Selbstsorge zu betonen.

Der Ansatz der Care-Ethik erweitert das Spektrum ethischer Verhaltensweisen, die pflegerischem Handeln zugrunde liegen, entsprechend in verschiedene Richtungen: hin zu Aufmerksamkeit und Verantwortung, Kompetenz und Resonanz. Zugleich wird die im ICN-Ethikkodex angeklungene ethische Verhaltensweise der Selbstsorge aufgenommen und substantiiert.

## Verhältnisbestimmung von Fürsorge und Selbstsorge

Die Betrachtung des ICN-Ethikkodex und der Prinzipienethik als Begründungsquelle für moralische Verantwortlichkeiten und Rechte von PP, erweitert um die Perspektive der Care-Ethik, ermöglicht es, einen ersten Versuch zu unternehmen, einen Korridor ethisch vertretbarer Pflege – sowohl für Patient*innen als auch für die PP selbst – aufzuzeigen. Dabei werden Für- und Selbstsorge nicht als sich gegenseitig ausschließend angesehen, sondern stehen komplementär zueinander. Im Vordergrund steht entsprechend nicht die Lösung eines Konflikts, sondern ein Abwägungsprozess. Die in diesem Zusammenhang auch relevante Abwägung zwischen Fürsorge für Patient*innen und Fürsorge für eigene Angehörige der PP kann hier nicht weiter verfolgt werden. Insbesondere im Kontext der Pandemie mit erhöhtem Arbeitsaufkommen und veränderten Rahmenbedingungen für die Versorgung (Schließung von Tagespflegeeinrichtungen für pflegebedürftige Angehörige, Kindergarten und -krippe; begrenzte Kapazitäten der Notfallbetreuung; Homeschooling) stellt sich die Frage, welcher Fürsorgepflicht Vorrang zukommt und in welchem Verhältnis hierzu die Dimension der Selbstsorge steht. Diese Frage kann jedoch hier nicht weiterverfolgt werden. Welche eindeutigen Grenzen des Korridors ethisch vertretbarer Pflege ICN-Ethikkodex, Prinzipienethik und Care-Ethik bei der Abwägung zwischen Selbstsorge und Fürsorge für Patient*innen vorgeben, wird im Folgenden in drei Schritten beleuchtet. Dabei widmen sich die ersten beiden der Abwägung von Selbst- und Fürsorge auf individueller Ebene (Mikroebene); der letzte zielt auf die übergeordneten Entscheidungsebenen (Meso- und Makroebene). Situationen, die innerhalb dieses Korridors anzusiedeln sind, können mithilfe der Instrumente (ICN-Ethikkodex, Prinzipienethik und Care-Ethik) nicht abschließend bewertet werden.

### Abwägung von Selbstsorge und Fürsorge auf der Mikroebene

In einem ersten Schritt stellt sich die Frage, ob einer Vernachlässigung der Selbstsorge eine Grenze gesetzt werden sollte und wie diese näher bestimmt werden kann. Nach dem ICN-Ethikkodex achtet die PP „auf ihre eigene Gesundheit, um ihre Fähigkeit zur Berufsausübung nicht zu beeinträchtigen“ (ICN 2012). Diese ethische Verhaltensweise der Gesunderhaltung ist direkt an die Fähigkeit zur Ausübung professioneller Pflege gebunden. Eine klare Grenze wird also dort gezogen, wo die Fürsorge für Patient*innen durch mangelnde Sorge der PP für ihr eigenes „psychisches, körperliches, soziales und geistiges Wohlbefinden“ (ICN 2012) nicht mehr adäquat, d. h. im Sinne des Kodex, ausgeführt werden kann. Daraus kann geschlossen werden, dass es dann nicht mehr als professionelle Verpflichtung angesehen werden kann, diese zu einer Beeinträchtigung führenden Sorgeaufgaben zu übernehmen, denn die Fürsorgepflicht Pflegender ist nicht absolut (McKenna 2020). Hier ist beispielsweise an den Fall zu denken, dass eine zur Risikogruppe gehörende PP bei Mangel an PSA auf einer COVID-19-Station eingesetzt wird. Da sie Kontaktperson ist, ist ihr zudem der monatliche ärztliche Kontrolltermin versagt. Zudem führt der Personalmangel zu einer erhöhten Arbeitsbelastung und aufgrund des damit einhergehenden Energie- und Zeitmangels einer weiter reduzierten Selbstsorge (Petzold et al. 2020). Bereits aus diesem instrumentellen Verständnis heraus folgt, dass Selbstsorge nicht komplett zugunsten der Fürsorge aufgegeben werden kann. Damit ist eine Grenze dort gezogen, wo vollständige „Selbstaufopferung“ und „Selbstaufgabe“ von PP gezeigt werden, wie dies ein immer noch zu findendes Pflegeverständnis nahelegt (DGP 2020), in dem Pflegenden vor allem aufgrund ihres „aufopferungsvollen Einsatzes“ (AEM 2020, S. 6) Anerkennung zukommt. Die mediale Darstellung von Pflegenden in „Helden-“ oder „Engel-Narrativen“ manifestiert derartige Stereotypen und untergräbt die pflegerische Professionalität (Stokes-Parish et al. 2020).

Die Care-Ethik erweitert diese Perspektive. Im Sinne der Reziprozität darf die Selbstsorge und damit Gesundheit der PP nicht rein als instrumentelles Gut betrachtet werden. Es gilt vielmehr, dass wirkungsvolles Caring auch die Verantwortung umfasst, die Sorgearbeit an Kolleg*innen zu übergeben, wenn die eigenen Grenzen erreicht sind, und eine solche Übergabe zu ermöglichen, bevor die völlige Erschöpfung eintritt (Bohlken et al. 2020). Insbesondere die Aussetzung oder Missachtung der PpU kann jedoch zu Fällen führen, in denen die PP ihre Grenzen und Bedürfnisse erkennt (attentiveness), die Sorgearbeit aber dennoch weiterhin ausführt oder ausführen muss. Exemplarisch seien verlängerte Schichten durch krankheitsbedingte Ausfälle in Kombination mit Personalmangel genannt. Für die PP liegt neben den Sachzwängen meist ein Gefühl der moralischen Verantwortung gegenüber Patient*innen und Kolleg*innen dahinter sowie das Erleben von moralischem Stress (Kohlen 2019; Knochel et al. 2020). Verstärkt werden kann dies zudem noch durch die von den Pflegenden wahrgenommene gesellschaftliche Vorstellung von „Pflege als Berufung“, die den moralischen Druck, selbstlos handeln zu müssen, noch verstärkt (Slettmyr et al. 2019). In Abwägung des care-ethischen, relationalen Verständnisses von Selbstsorge und der Prinzipien der Fürsorge sowie des Nicht-Schadens lässt sich dementsprechend zwar festhalten, dass sich Selbstsorge in Ausnahmesituationen zeitweise der Fürsorge unterordnen *kann*, dies allerdings nur soweit wie weiterhin wirkungsvolle, verantwortete Sorgearbeit erbracht werden kann.

Im anderen Extrem, und damit in Annäherung an den zweiten Eckpfeiler auf individueller Ebene, kann die Situation auftreten, dass PP – ohne Vorliegen anderer Gründe wie einer Vorerkrankung oder informeller Sorgearbeit – den Schutz und die Sorge ihrer selbst über die Fürsorge von Patient*innen stellen. Insbesondere auf den Einsatz dieser PP dürfte eine Zwangsrekrutierung, wie diese zeitweise angedacht war (vgl. Epidemie-Gesetzentwurf NRW und Niedersachsen), abzielen, auf deren ethische Vertretbarkeit als solche vorliegend nicht eingegangen werden kann. Der Überordnung von Selbstsorge über Fürsorge für Patient*innen jedoch steht nicht nur die professionelle Verantwortung gegenüber den pflegebedürftigen Menschen, sondern auch die „mit der Gesellschaft [geteilte] Verantwortung“ entgegen, „Maßnahmen zugunsten der gesundheitlichen […] Bedürfnisse der Bevölkerung […] zu unterstützen“ (ICN 2012, S. 2). Es ist gerade auch für die Care-Ethik und ihren Fokus auf Relationalität charakteristisch, dass sie sich nicht auf die Beziehung zwischen PP und Patient*in beschränkt, sondern das Netzwerk sozialer Beziehungen im Blick hat, von welchem PP und Patient*innen Teil sind. Entsprechend erstreckt sich auch die Aufmerksamkeit auf dieses Netzwerk und wäre das Ignorieren der Bedürfnisse der Patient*innen und Kolleg*innen ein moralisches Übel. Eine Priorisierung eigener Bedürfnisse würde zudem bedeuten, auf die Bedürfnisse anderer, trotz vorhandener Kompetenz, nicht adäquat zu reagieren. Daraus resultiert also, dass eine *kategorische* Unterordnung von Fürsorge unter Selbstsorge ethisch nicht zu rechtfertigen ist.

### Abwägung von Selbstsorge und Fürsorge auf der Meso- und Makroebene

Die Verhältnisbestimmung von Selbst- und Fürsorge erfolgt nicht nur individuell, sondern ist bereits in den Maßnahmen und politischen Empfehlungen selbst eine Abwägung impliziert. Der ICN-Ethikkodex selbst kann bezüglich des politisch ausgearbeiteten „crisis standard of care“ als solchem keine eindeutige Antwort für die Verhältnisbestimmung zwischen Selbst- und Fürsorge liefern, da er ausschließlich ethische Verhaltensweisen für PP formuliert, nicht aber politische Institutionen bindet. Auch die Prinzipienethik fokussiert sich auf die Patient*in-Gesundheitsprofession-Beziehung. In diesen Fällen erlaubt die Care-Ethik als kritisches Analyseinstrument politischer Entscheidungen eine übergeordnete Verhältnisbestimmung von Selbst- und Fürsorge (Tronto 1993, S. 172) und kann damit komplementär zu Gerechtigkeits- und Menschenrechtstheorien auch für Abwägungen auf Public Health Ebene fruchtbar gemacht werden. Denn neben den Patient*innen haben auch die PP selbst als ebenso vulnerable, interdependente Mitglieder der Gesellschaft im Fokus des staatlichen „care-about“ und der (sozial-)staatlichen Verantwortung zu stehen. Die vorgestellten Maßnahmen stehen zwar im- oder gar explizit unter dem Diktum „Entlastung der Pflegekräfte“ (Bundesregierung 2020), sind jedoch vor dem Hintergrund einer Abwägung von Für- und Selbstsorge kritisch zu beleuchten. Zum einen folgern sie aus PSA- und Personalmangel als *empirischen* Problemen eine Herabsetzung der Hygienemaßnahmen und Quarantäne-Zeit. Inwieweit eine derartige Anpassung von Empfehlungen, die im Sinne der Care-Ethik auch eine moralische Qualität haben, ein naturalistischer Fehlschluss von Sein auf Sollen darstellt (eine Ableitung der Anpassung normativer Standards aus einer empirischen Mangelsituation), müsste für die Maßnahmen einzeln beleuchtet werden. Zum anderen, und für den vorliegenden Beitrag bedeutender, ist fraglich, ob sich der „crisis standard of care“ als solcher im ethisch vertretbaren Korridor von Für- und Selbstsorge bewegt.

Ein Mangel an PSA, Verkürzung der Quarantäne-Zeit und personelle Unterbesetzung stellen gerade auf ITS nicht nur eine potenzielle Gefährdung für PP und das gesamte Team dar, sondern gehen mit einer erhöhten (Infektions‑)Gefahr, reduzierter Pflegequalität und geringerer Patient*innensicherheit einher (Ausserhofer et al. 2014; Schmitz-Rixen und Grundmann 2020). Dies stellt PP vor ein doppeltes Dilemma: Auf der einen Seite werden sie in der Ausübung der aus dem ICN-Ethikkodex und der Care-Ethik resultierenden ethischen Verhaltensweise der Selbstsorge beschnitten. Auf der anderen Seite wird ihnen zudem verwehrt, ihrer professionellen Verantwortung – oder gar Pflicht – der Fürsorge unter Einhaltung pflegepraktischer Standards nachzukommen. Der für die Mikro-Ebene herausgearbeitete Korridor ist deshalb um die Sorge des Staates auch für das Wohl der PP als moralischer Akteure zu komplementieren. Es folgt entsprechend, dass auch staatliche Akteure die Selbstsorge der PP (sowie deren Fürsorge für An- und Zugehörige) nicht *komplett* zugunsten der Fürsorge für Patient*innen unterminieren dürfen. Die weitergehende Frage, inwieweit und in welchen Fällen staatliche Stellen PP dazu verpflichten können, ihre Selbstsorge (und Fürsorge für ihre An- und Zugehörigen) der Fürsorge für Patient*innen unterzuordnen, erfordert eine umfassende Abwägung unter Gesichtspunkten wie Solidarität, Gerechtigkeit und Verantwortung, welche hier nicht geleistet werden kann.

## Conclusio

Auch wenn an dieser Stelle kein umfassendes Bild gezeichnet werden kann, ergeben sich folgende Eckpfeiler als Antwort auf die Frage, wie sich in der COVID-19 Pandemie Fürsorge für Patient*innen und Selbstsorge von ITS-PP zueinander verhalten *sollten*:

1. Fürsorge darf auch in der besonderen Pandemiesituation *nicht regelhaft* der Selbstsorge übergeordnet werden – weder durch die PP selbst noch durch politische und andere Entscheidungsträger*innen.
2. Eine Unterordnung von Selbstsorge unter Fürsorge kann jedoch *zeitweise*, im Rahmen einer derartigen Ausnahmesituation – auf der Basis einer intra-individuellen Abwägung von PP, nicht auf der Basis einer Verpflichtung durch Dritte – erfolgen.
3. Allerdings ist, umgekehrt, eine *kategorische* Unterordnung von Fürsorge unter Selbstsorge ethisch nicht zu rechtfertigen.

Diese Eckpfeiler können nicht mehr als Grenzen aufzeigen, die eine Hilfestellung für die praktische Implementierung des Selbst- und Fürsorgegedankens für PP auf ITS bieten, jedoch den je konkreten Aushandlungsprozess, abhängig von der Situation auf der konkreten ITS und den Bedürfnissen der PP, nicht ersetzen können. Deutlich wurde in der Abwägung jedoch, dass Selbstsorge ein *essentieller* Bestandteil der Sorgearbeit ist. Das Hervorheben von Selbstsorge darf nicht darüber hinwegtäuschen, dass diese ebenso relational zu verstehen ist und folglich die Sorge um das Wohl der Pflegebedürftigen mit einbezieht. Für Deutschland fehlt bislang belastbare Evidenz für den Einfluss des „crisis standard of care“ auf die Gesundheit von ITS-PP. Solche Evidenz zu generieren und ein Raster für die Abwägung im Einzelfall zu entwickeln, welches auch die jeweilige Selbstreflexionsfähigkeit der PP berücksichtigt, bleibt ein Desiderat der Forschung. Schließlich changieren die Eckpfeiler zwischen Mikro- und Makroebene, und wird auf die Verteilung und Zuschreibung von Verantwortung innerhalb eines Krankenhauses nicht explizit eingegangen. Dies resultiert aus der Natur der in der COVID-19 Pandemie verabschiedeten Gesetze und politischen Empfehlungen, die wenig Handlungsspielraum lassen und stark in die Selbstverwaltung eingreifen.

Dennoch kann gerade auf der Mesoebene über die Implementierung eines wertorientierten Pflege-Managements, das Selbstsorge als Teil der professionellen Grundhaltung von PP anerkennt und diese aktiv unterstützt, relationale Selbstsorge kultiviert werden. Zudem ist jedoch ein umgreifender Prozess auf übergeordneter Ebene notwendig und kommt hier politischen wie gesellschaftlichen Akteuren im Sinne der Care-Ethik auch eine Verantwortung dafür zu, die instrumentelle wie relationale Selbstsorge der PP in zukünftiger „pandemic prepardness“ mitzudenken und nicht durch Aussetzen von PpU oder Empfehlungen zu einem Weiterarbeiten bei asymptomatischer Infektion vollständig zu unterminieren: Wie das ICN, an die PP gerichtet, feststellt, ist „[d]er bisherige Verlauf des Internationalen Jahres der Pflegenden (…) nicht das Jahr, das wir geplant hatten. Aber durch Ihre Arbeit, durch Ihr Handeln, durch Ihr Pflegen zeigen Sie so deutlich, warum professionell Pflegende mehr Anerkennung, mehr Investitionen und mehr Unterstützung brauchen“ (ICN 2020a). Hierzu gehören eine ausreichende Personalausstattung mit PP (DBfK 2020c), eine angemessene Berücksichtigung persönlicher Gesundheit und Sicherheit der PP (Fowler 2018) und öffentliche Wertschätzung – folglich Rahmenbedingungen, die einer der Pflegeprofession inhärenten Notwendigkeit der stetigen Verhältnisbestimmung zwischen Fürsorge und Selbstsorge gerecht werden.

## Footnotes

1 Im Folgenden wird durchgehend von „Pflegefachpersonen“ gesprochen. Gemeint sind damit die dreijährig ausgebildeten, staatlich examinierten Pflegeberufe.

2 Die Situation stellt sich zugespitzt auf ITS dar. Die Autorinnen möchten jedoch ausdrücklich darauf hinweisen, dass die geschilderten Problemlagen auch für alle anderen klinischen und ambulanten pflegerischen Arbeitsbereiche zutreffen.

3 Eine ausführliche Betrachtung der Situation beruflicher Pflege unter sozialethischen Gesichtspunkten findet sich bei Bobbert (2019).

4 Lt. Gesetzgeber sind dies Arbeitsbereiche, in denen es empirische Nachweise dafür gibt, dass sich eine Unterbesetzung mit PP ungünstig auf das Patient*innenoutcome im Hinblick auf unerwünschte Ereignisse wie Infektionen und Dekubitus auswirkt.

5 Vgl. dazu „Wiederverwendung von MNS und FFP2- und FFP3-Masken“ (RKI 2020a, S. 1).

6 Das RKI berichtet (Stand: 17.12.2020) von 37.080 COVID-19 Fällen bei Personal in medizinischen Einrichtungen nach §23 des Infektionsschutzgesetzes (https://www.rki.de/DE/Content/InfAZ/N/Neuartiges_Coronavirus/Situationsberichte/Dez_2020/2020-12-17-de.pdf?__blob=publicationFile, zugegriffen: 18. Dez. 2020).

7 Zu der kontrovers diskutierten Frage, ob Ethik in der Pflege und für die Pflege als sog. „Pflegeethik“ eine eigene Bereichsethik ist oder zu der Medizinethik zu zählen ist, siehe bspw. Bobbert (2003) und Rehbock (2000).

8 Eine Überarbeitung des ICN-Ethikkodex wurde am 23. Mai 2019 angekündigt (Stievano und Tschudin 2019).

9 Bspw. https://www.dbfk.de/de/presse/meldungen/2018/1754843991.php (zugegriffen: 13.04.2020).

10 Siehe https://deutscher-pflegerat.de/pflegeberuf/berufsethik.php (zugegriffen: 13.04.2020).

11 § 5 Abs. 2 S. 2 des Pflegeberufereformgesetzes formuliert explizit unter der Überschrift Ausbildungsziel: „[Pflege] erfolgt entsprechend dem allgemein anerkannten Stand pflegewissenschaftlicher, medizinischer und weiterer bezugswissenschaftlicher Erkenntnisse auf Grundlage einer professionellen Ethik.“ Siehe auch Lehmeyer und Riedel (2019).
